# Supplementary material for: SiO2-Ag Composite as a Highly Virucidal Material: A Roadmap that Rapidly Eliminates SARS-CoV-2
Source: Nanomaterials (Basel). 2021 Mar 4;11(3):638. doi: 10.3390/nano11030638 (PMC8001031; doi:10.3390/nano11030638)
Supplement: Supplementary file 1 [file nanomaterials-11-00638-s001.pdf]

nanomaterials

Supplementary Material

SiO_2-Ag Composite as a Highly Virucidal Material: A Roadmap That Rapidly Eliminates SARS-CoV-2

Marcelo Assis[1,2], Luiz Gustavo P. Simoes[3], Guilherme C. Tremiliosi[3], Dyovani Coelho[1], Daniel T. Minozzi[3], Renato I. Santos[3], Daiane C. B. Vilela[3], Jeziel Rodrigues do Santos[1], Lara Kelly Ribeiro[1], Ieda Lucia Viana Rosa[1], Lucia Helena Mascaro[1], Juan Andrés[2,*] and Elson Longo[1]

[1] CDMF, LIEC, Federal University of São Carlos - (UFSCar), 13565-905 São Carlos, SP, Brazil. marcelostassis@gmail.com (M.A.); dyovani@gmail.com (D.C.); prof.jeziel@gmail.com (J.R.S.); larakribeiro@gmail.com (L.K.R); ilvrosa@ufscar.br (I.L.V.R.); lmascaro@ufscar.br (L.H.M.); elson.liec@gmail.com (E. L.)

[2] Department of Physical and Analytical Chemistry, University Jaume I (UJI), Castellon 12071, Spain.

[3] Nanox Tecnologia S/A, 13562-400 São Carlos, SP, Brazil. gustavo@nanox.com.br (L.G.P.S.); guilherme@nanox.com.br (G.C.T.); daniel@nanox.com.br (D.T.M); renato.santos@nanox.com.br (R.T.S); microbiologia@nanox.com.br (D.C.B.V)

* Correspondence: andres@qfa.uji.es

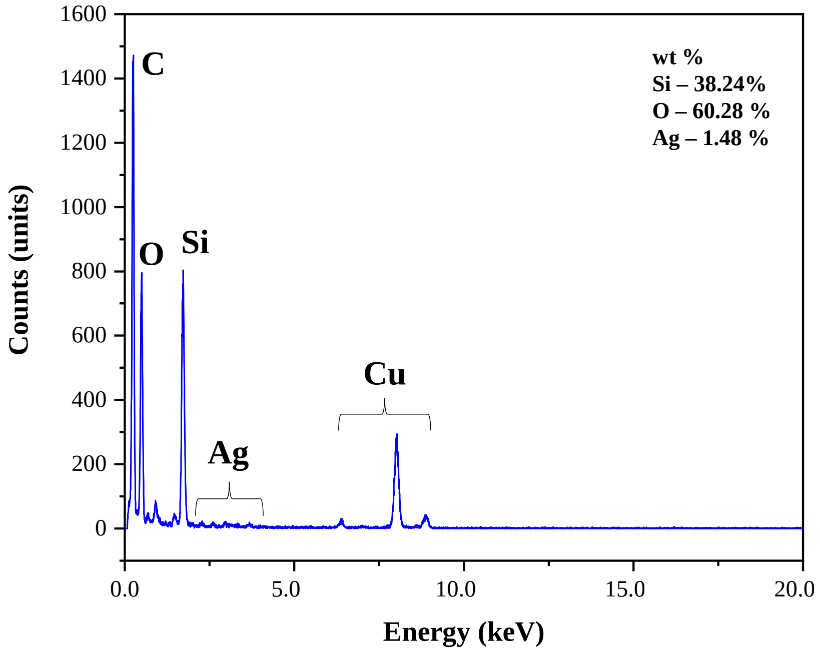

Figure S1. Chemical composition from EDX analysis of the SiO_2-Ag.

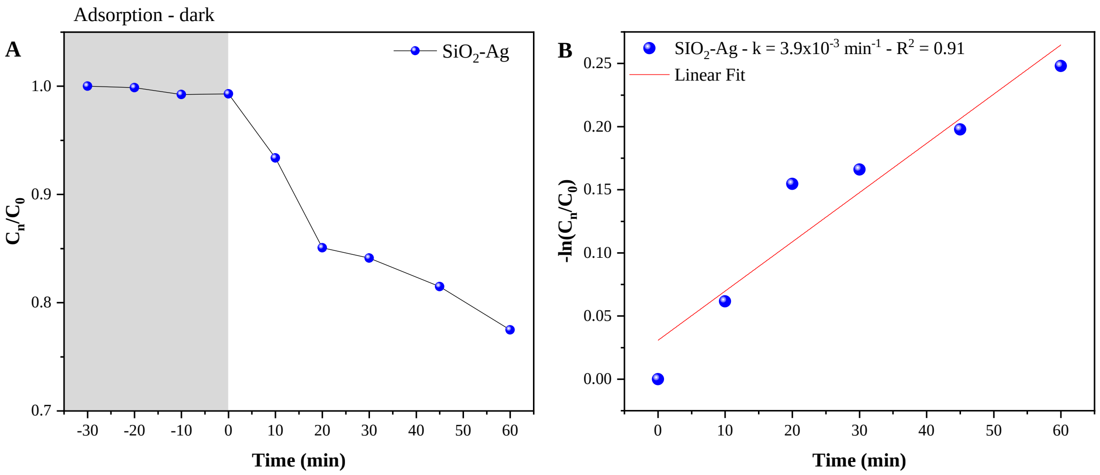

Figure S2. (**A**) Relative concentration of RhB dye (C_n/C_0). (**B**) Reaction kinetics of the RhB degradation $-\ln(C_n/C_0)$ versus time (min) for SiO_2-Ag composite.

Silica surfaces have been extensively studied through theoretical calculations with both a cluster and periodic approach. The choice of the modeling method is still a matter of debate, since studies show that cluster calculations that do not take into account the long-range interaction approximations imposed by the extremities are not adequate to describe the silanol groups (Si-OH). For our model we have 1 ring with 4 silicon, 5 silicon and 7 silicon. Our model is composed by three polygons: square, pentagon and heptagon, and later the atoms with the same position were removed. (Figure S3) So this model has 12 Si atoms and 22 oxygen atoms, that is, the surface has two oxygen vacancies. The corresponding optimized SiO_2 model was presented in Figure S4 and Table S1. On the other hand, it is well known that the functional hybrid B3LYP is appropriate for studies of interactions with small molecules, such as H_2O. [1,2]

Figure S3 Schematic representation of the different rings used for modeling SiO_2. Silicon (yellow) and Oxygen (red).

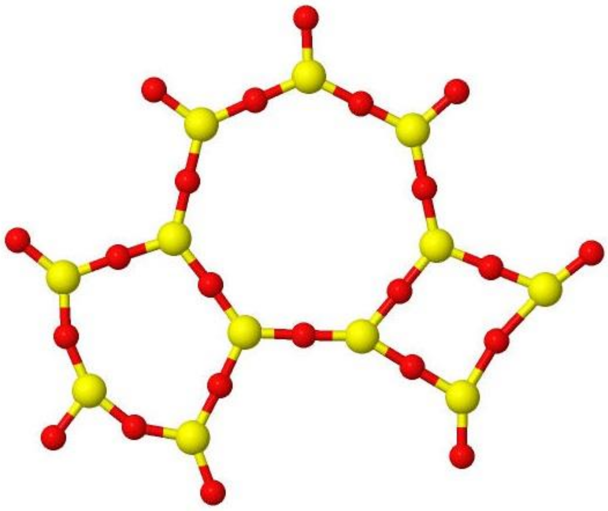

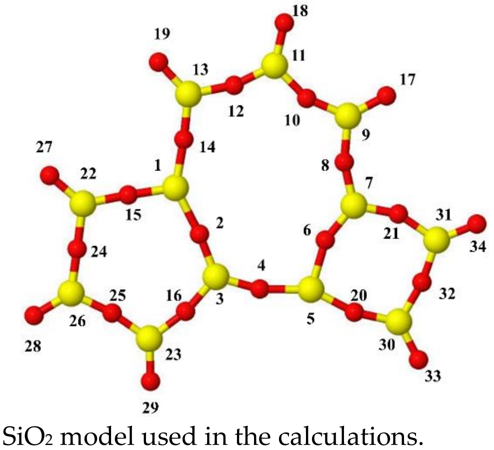

Figure S4. The optimized SiO_2 model used in the calculations.

Table S1 Bond angles and lengths of the structure used.

connectivity	bond length (Å)	connectivity	bond angle (º)
Si_1-O_2	1.86	O_{14}-Si_1-O_2	143.2

O_2-Si_3	1.65	O_2-Si_3-O_4	126.5
Si_3-O_4	1.61	O_4-Si_5-O_6	105.1
O_4-Si_5	1.81	O_6-Si_7-O_8	127.2
Si_5-O_6	1.85	O_8-Si_9-O_{10}	109.1
O_6-Si_7	1.60	O_{10}- Si_{11}-O_{12}	108.1
Si_7-O_8	1.59	O_{12}- Si_{13}-O_{14}	106.8
O_8-Si_9	1.64	O_{15}- Si_1-O_2	108.4
Si_9-O_{10}	1.60	O_{16}- Si_3-O_2	117.7
O_{10}-Si_{11}	1.63	O_{25}-Si_{23}-O_{16}	107.8
Si_{11}-O_{12}	1.60	O_{24}-Si_{26}-O_{25}	108.2
O_{12}-Si_{13}	1.64	O_{24}- Si_{22}-O_{15}	108.9
Si_{13}-O_{14}	1.61	O_{20}-Si_6-O_5	101.6
O_{14}-Si_1	1.74	O_{21}-Si_7-O_6	119
O_{15}-Si_1	1.70	O_{32}-Si_{30}-O_{20}	107.3
O_{16}-Si_3	1.63	O_{32}-Si_{31}-O_{21}	105.6
O_{17}-Si_9	1.54	O_{17}-Si_9-O_8	122.2
O_{18}-Si_{11}	1.54	O_{18}-Si_{11}-O_{10}	123.1
O_{19}-Si_{13}	1.54	O_{19}-Si_{13}-O_{12}	122.3
O_{20}-Si_5	1.74	O_{27}-Si_{22}-O_{15}	128.5
O_{21}-Si_7	1.60	O_{28}-Si_{26}-O_{24}	128.8
Si_{22}-O_{15}	1.62	O_{29}-Si_{23}-O_{25}	126.5
Si_{23}-O_{16}	1.62	O_{33}-Si_{30}-O_{20}	130.3
O_{24}-Si_{22}	1.62	O_{34}-Si_{32}-O_{33}	130.0
O_{25}-Si_{23}	1.62	-	-
Si_{26}-O_{25}	1.62	-	-
O_{27}-Si_{22}	1.54	-	-
O_{28}-Si_{26}	1.54	-	-
O_{29}-Si_{23}	1.54	-	-
Si_{30}-O_{20}	1.63	-	-
Si_{31}-O_{21}	1.66	-	-
O_{32}-Si_{30}	1.64	-	-
O_{33}-Si_{30}	1.54	-	-
O_{34}-Si_{31}	1.54	-	-

In Figure S5 the map of the molecular electrostatic potential of the SiO_2 is displayed. The regions acting as electron receptor for H_2O and an electron donor for molecular O_2 are highlighted. The analysis of the charge transfer from H_2O to SiO_2 is 0.04 e^-, on the other hand, the surface of SiO_2 is able to transfer 0.10 e^- to O_2 molecule After this charge transfer, new ectrophilic/nucleophilic centers appear on the surface giving the quantum probability of new interactions.

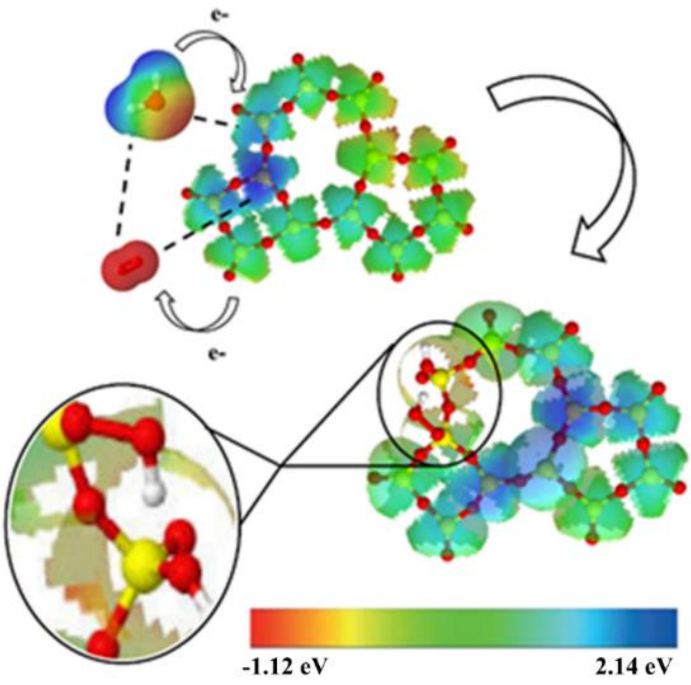

Figure S5 MEP (in eV) of SiO_2 model.

References

1. Civalleri, B.; Garrone, E.; Ugliengo, P. Ab Initio Study of the Adducts of Small Molecules with the Isolated Hydroxyl of Silica and the Brønsted Site in Zeolites: A Comparison between B3-LYP and MP2 Methods. *J. Phys. Chem. B* **1998**, *102*, 2373–2382, doi:10.1021/jp972281b.
2. Bera, S.; Won, D.-I.; Rawal, S. B.; Kang, H. J.; Lee, W. I. Design of Visible-Light Photocatalysts by Coupling of Inorganic Semiconductors. *Catal. Today* **2019**, *335*, 3–19, doi:10.1016/j.cattod.2018.11.001.
